# A Comparative Study of the Thermal Conductivities of CBA Porous Concretes

**DOI:** 10.3390/ma15155204

**Published:** 2022-07-27

**Authors:** Seung-Tae Jeong, Quang-The Bui, In-Hwan Yang

**Affiliations:** Department of Civil Engineering, Kunsan National University, Kunsan 54150, Korea; stjeong@kunsan.ac.kr (S.-T.J.); tqbui93@gmail.com (Q.-T.B.)

**Keywords:** porous concrete, coal bottom ash aggregate, thermal conductivity, porosity, unit weight

## Abstract

Porous concrete has recently gained increasing attention in the construction industry. To improve the properties of porous concrete, coal bottom ash (CBA) was used as the aggregate in the concrete mixtures studied herein. Hybrid CBA aggregates, including a 20% proportion of particles with sizes of 1.2~2.5 mm and an 80% proportion of particles with sizes of 2.5~5.0 mm, were used in the mixtures. Various water/cement ratios ranging from 0.25 to 0.35 were used in the mixtures. The effects of compaction at 0.5, 1.5, and 3.0 MPa on the properties of the porous concrete were also examined. The increase in the water/cement ratio reduced the unit weight and thermal conductivity while increasing the porosity of the porous concrete. Although the compaction had a significant impact on the other properties of the porous concrete, the thermal property was not significantly influenced. By using CBA in porous concrete, the mechanical and thermal properties of the concrete were significantly improved. Finally, the relationships between the thermal conductivity and other properties of the porous concrete were investigated.

## 1. Introduction

Developing novel materials with multiple properties that benefit the environment and support sustainable development is a global trend. Among the novel materials, porous concrete has received increasing attention in the civil construction field because it exhibits a wide range of applications [1,2,3]. It is well known that porous concrete forms structures with high porosity, and thus, it can be applied as acoustic noise absorbing or thermal insulation materials and used in permeable water facilities. Annually, it is estimated that approximately 730 million tons of CBA are collected throughout the world from thermal power plants [4]. In South Korea, it is reported that the amount of CBA collected from thermal sources is approximately 9 million tons per year [5]. With the development of these technologies, CBA can be used in various fields, such as metal recovery, material synthesis, wastewater treatment, soil amelioration, catalysis, the ceramic industry, and particularly in the construction industry [6]. In the construction industry, CBA can be used to replace the cementitious material of fine and coarse aggregates. Muthusamy et al. [7] reviewed the usage of CBA in concrete and concluded that the use of CBA in concrete benefits the environment and economy. In addition, this study pointed out that the combination of CBA in concrete reduces the unit weight of concrete, but in the long term, the strength, as well as the durability, would be improved by the pozzolanic reaction from CBA.

The properties of porous concrete result from the volume of open or interconnected voids. Therefore, to improve these properties, the utilization of lightweight coarse aggregates, to which little or no sand is added, and an adequate amount of cement paste is recommended for porous concrete [3]. Zaetang et al. [8] reported that the insulating properties of porous concrete were increased by three to four times when lightweight coarse aggregates were used. Recently, coal bottom ash (CBA) has been progressively used in the construction industry due to its ability to replace cement or aggregate in concrete [9,10]. According to previous studies [9,11,12,13], the CBA particles contain micro- and macro-pores that make the density of CBA low. Zhou and Brooks [14] studied the influences of lightweight aggregates on the thermal and mechanical properties of lightweight concrete. They found that the use of lightweight aggregate significantly reduced the density and thermal properties of the concrete. Yang and Park carried out a study on the thermal properties of concrete containing CBA [15]. Their test results revealed that the thermal conductivity of the concrete was reduced with the increasing CBA content. For this reason, using CBA as a replacement for aggregate in porous concrete would reduce the unit weight and thermal conductivity of the concrete. Ngohpok et al. [16] reported that the unit weight of porous concrete was decreased by up to 38.8% when CBA was used in the mixtures. Jang et al. [17] investigated the physical properties of porous concretes containing CBA as a coarse aggregate, and low unit weights of porous concrete with geopolymers were observed.

The physical properties of porous concrete mainly depend on those of the cement paste and aggregates used, and thus, the water/cement ratio may also have an effect on the properties of the porous concrete. Huang et al. [18] conducted a study on porous concrete and sought to optimize the mixture for the production of porous concrete. The test results indicated that the optimal water/cement ratio for the porous concrete was in the range of 0.25 to 0.30. Kováč and Sičáková also found that water/cement ratios in the range of 0.25 to 0.35 influenced the key properties of porous concrete [19]. They reported that the water/cement ratio had an insignificant effect on the strength of porous concrete. To investigate the effects of cement paste on the properties of porous concrete, Li et al. [20] fabricated porous concrete with various water/cement ratios. Based on the test results, they recommended a water/cement ratio of 0.25 as the optimal value for the porous mixture.

Porous concrete contains only coarse aggregate or a small amount of fine aggregate and a low water-cement ratio, and thus the cement paste and aggregate act as the skeleton of the porous concrete. Therefore, the workability of porous concrete is lower than that of conventional concrete, and compaction of the concrete may be required to improve the adhesion between the cement paste and coarse aggregate.

According to a study by Bonicelli et al. [21], compaction energy levels affected the mechanical characteristics of porous concrete. In their study, four different compacting energy levels were applied. They found that the unit weight of the porous concrete was increased, but its porosity was reduced when high compaction energy levels were applied. In an effort to develop porous concrete in cold weather climates, Schaefer et al. [22] also cast porous concrete with regular and low compaction energy levels. Their test results revealed that the mechanical properties of the porous concrete were significantly influenced by the compaction energy levels. Additionally, it is recommended that more studies be conducted to determine the relationships between the compaction energy and the mechanical properties of porous concrete.

The addition of small amounts of fine aggregates to lightweight concrete significantly improved the mechanical properties of porous concrete [21]. However, the addition of fine aggregates to porous concrete reduced its drainability [21,23]. Barnhouse and Srubar [24] reported that the porosity of porous concrete was reduced by 16% when sand was used as a coarse aggregate replacement of 7% by mass. In addition, the world is facing a sand shortage [25], and it is reported that over 50 million tons of sand were mined in developed countries, such as the United States, China, and Australia. Hence, limiting the use of sand or finding alternative materials for sand is necessary. The thermal conductivities of lightweight concrete containing CBA and sand were also investigated by Yang et al. [26]. In this study, the addition of sand to the lightweight concrete caused the macrostructure to become denser and, thus, led to an increase in the unit weight of the concrete. Previous studies implied that porous concretes might require a high void content to improve acoustic noise absorption, thermal insulation, or water permeability characteristics. Therefore, it is necessary to investigate the thermal conductivities of porous concretes that do not include sand.

This study was designed to investigate the effects of the water/cement ratio, and the compaction level on the thermal conductivities of hybrid CBA porous concretes made without sand. The unit weights, porosities, and thermal conductivities of the CBA porous concrete were investigated. In addition, the relationships between the thermal conductivity and the physical properties of the CBA porous concrete were analyzed.

## 2. Experimental Details

**Figure 1:**
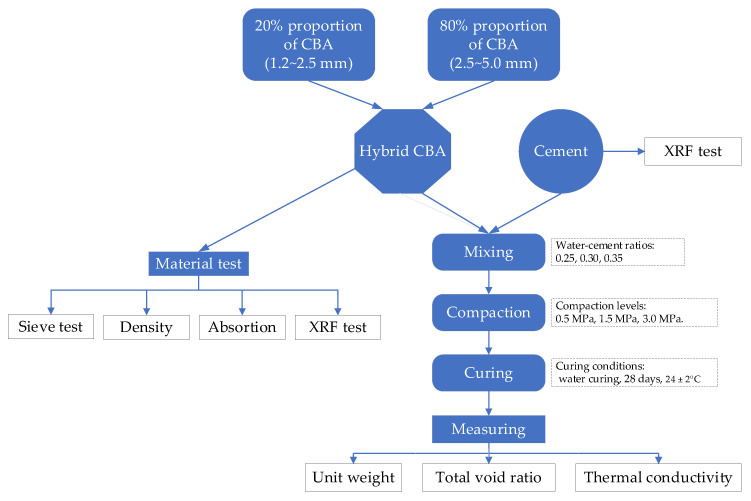
Full-scale test flowchart.

The full-scale experiment used in this study is illustrated in Figure 1.

### 2.1. CBA Aggregate and Cement

**Figure 2:**
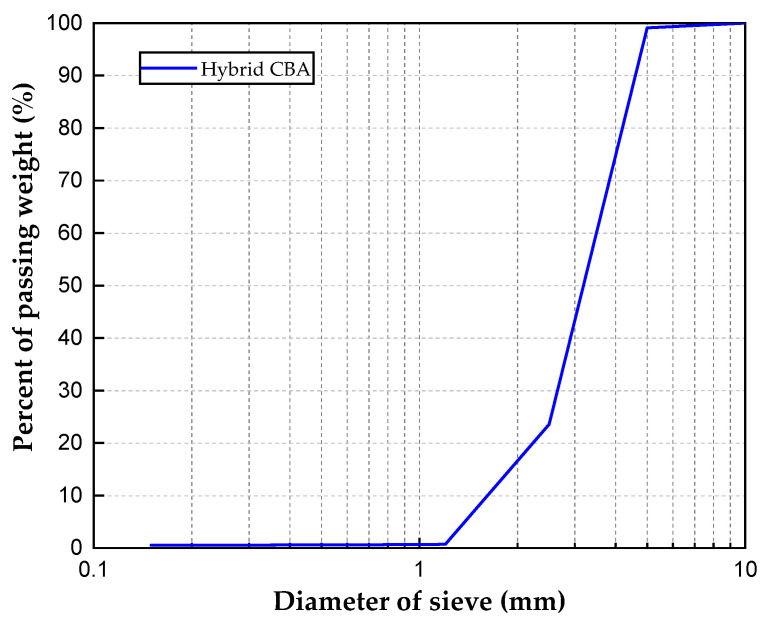
Results of sieve analyses for hybrid CBA.

The CBA used in the mixtures was a hybrid aggregate with two types of CBA with different particle sizes. The hybrid aggregate included an 80/20 volume percent ratio of CBA with a size range of 2.5–5.0 mm and CBA with a size range of 1.2–2.5 mm. The CBA was obtained from a thermal power plant company named Korea South-East Power Co., Ltd., located in Yeongheung, Korea. These large original CBAs were ground with a ball mill to obtain the desired sizes. In this study, fine aggregates were not included in the mixtures because the addition of fine aggregates reduced the porosity, drainability, and thermal isolation properties of the porous concrete [27]. To achieve such a small size of the CBA would require a large amount of time for grinding and increase the price of the CBA porous concrete. Therefore, the sizes and proportions of the CBA were determined for the mixtures. The percentages of the CBA sizes were determined by performing sieve tests in accordance with Korean standard KS F 2502 [28], as shown in Table 1. Figure 2 shows the particle size distribution of the hybrid CBA. This implies that the particle sizes of the hybrid CBA were distributed between 1.2 and 5.0 mm. The physical properties of the hybrid CBA are shown in Table 2. For the saturated surface dry (SSD) state, a low density of the hybrid CBA was indicated by a value of 1.73 g/cm^3^. For the oven-dried state, the hybrid CBA presented a lower density of 1.61 g/cm^3^. The table also shows that water absorption by the hybrid CBA was very high, with a value of 7.75%.

The cement used in the mixtures was ordinary Portland cement type I (OPC), which was provided by the Ssangyong C&E Company located in Jung–gu, Seoul, Korea. The density and the fineness modulus of OPC were 2.23 g/cm^3^ and 3650 cm^2^/g, respectively. Table 3 presents the chemical compositions of OPC.

Table 3 presents the chemical compositions of CBA. The X-ray fluorescence (XRF) spectrometry analysis results show that the CBA used in this study included high amounts of SiO_2_ (55.75%) and Al_2_O_3_ (26.15%). It was reported that the high amounts of these components improved the pozzolanic reaction of the concrete.

### 2.2. Mixtures

Nine mixtures were prepared to investigate the effects of the water/cement ratio and compaction on the thermal conductivities of the CBA porous concrete. The details for each mixture are shown in Table 4. The mixtures are identified by the water/cement ratio and the compaction level. The first numbers in the mixture labels represent the compaction levels of 0.5, 1.5, and 3.0 MPa, respectively. The last numbers represent the water/cement ratios of 0.25, 0.30, and 0.35, respectively.

Due to its high-water absorption capacity, the hybrid CBA was prepared under SSD conditions to provide the water needed for hydration. In addition, a cohesive agent was added to the mixtures to improve the adhesion between the aggregate particles.

### 2.3. Casting and Curing Conditions

Porous concrete is less consolidated than other concrete because it does not contain sand, which results in a porous structure for the concrete. Therefore, compaction is necessary to enhance the adhesion between the aggregates in porous concrete. In this study, special molds with thicknesses of 10 mm were prepared to secure the shapes of concrete specimens during the compaction loading. The porous concrete was cast in a steel cylindrical mold with dimensions of 100 mm (diameter) × 230 mm (height).

**Figure 3:**
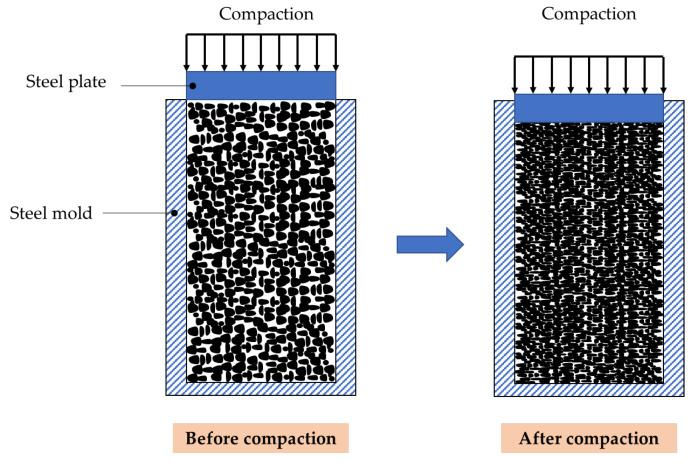
Schematic drawing for compaction of a cylindrical concrete specimen.

**Figure 4:**
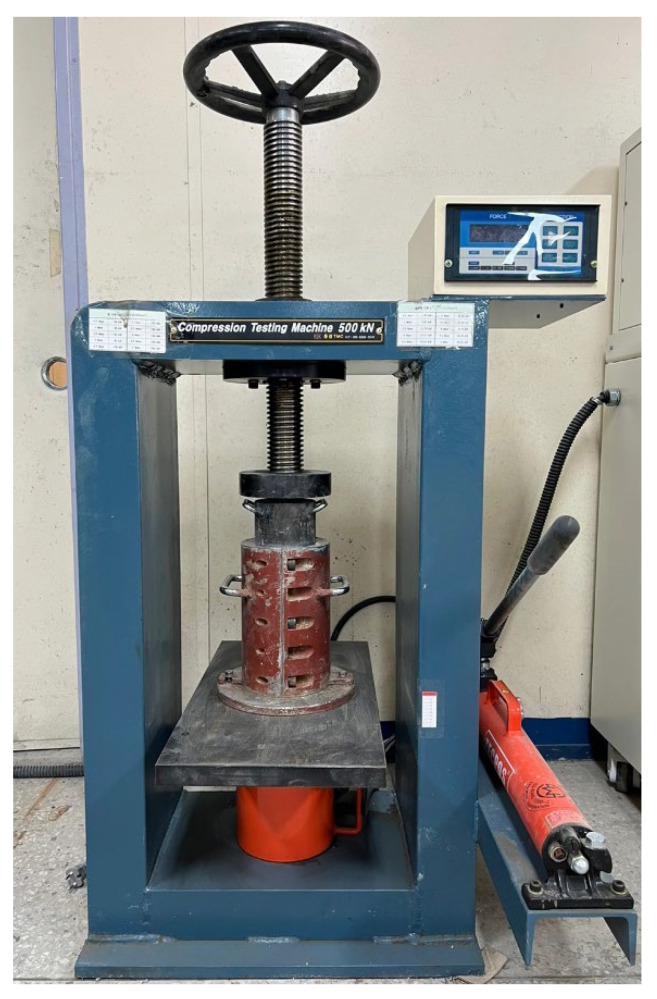
Actual compaction of a cylindrical concrete specimen.

The schematic compaction and steel cylindrical mold are shown in Figure 3. The top surfaces of the cast concrete specimens were compacted by a steel plate that was controlled by a hydraulic pump, as shown in Figure 4.

After 24 h of casting, the concrete specimen was demolded and then cured in a water tank at 24 ± 2 °C for 28 days in accordance with KS F 2405 [29].

### 2.4. Testing Methods

The unit weights, porosities, and thermal conductivities of the CBA porous concrete samples were measured in this study. The unit weight and porosity of the porous concrete were measured with cylindrical specimens, which were in accordance with Korean Standard KS F 2405 [29].

To evaluate the unit weight of the porous concrete, the lengths and diameters of three cylindrical specimens were carefully measured, and then each porous specimen was weighed. Finally, the unit weight of the porous concrete was calculated.

To determine the porosity characteristics of the CBA porous concrete, the total void ratio of the concrete was analyzed. The masses of the porous concrete specimen in water (*M_w_*) and in the dry state (*M_d_*) were obtained. The *M_w_* value was measured when the cylindrical specimen was totally immersed in the water. Due to the buoyant force in the water, the *M_w_* value is lower than the *M_d_* value. For the dry condition, the porous specimens were dried at 105 ± 5 °C for 24 h. Finally, the total void ratios of the porous specimens were determined as follows:

(1) $$V_{t}(\%)=1-\left(\frac{M_{d}-M_{w}}{\rho_{w}\times V}\right)\times 100$$

where *V_t_* is the total void ratio, $\rho_{w}$ is the density of water, and *V* is the volume of the cylindrical specimen. The reduced weight of the specimen in the water is equivalent to the weight of the water displaced by the CBA. Therefore, the total void ratios can be calculated by using the above equation.

The thermal conductivity of the porous concrete was estimated according to ASTM D 5334-05 [30] by applying the transient plane source (TPS) method [31,32]. The TPS1500 testing device used in this study was provided by Hot Disk, Ltd. (Gothenburg, Sweden), and a hot disk sensor was contacted between the halves of two porous concrete specimens. The hot disk sensor was heated during the operation of the TPS1500 device, and then the thermal conductivity of the porous concrete was estimated. To obtain the precise thermal conductivity of the CBA porous concrete, the measurement of each specimen was carried out within 30 min under room conditions. During the measurement process, the temperature rose *T(t)* near the hot disk’s surface, and the nondimensional function of time *D(t)* was recognized. Then, a linear relationship between *T(t)* and *D(t)* was estimated, and the slope of this line determined the thermal conductivity, *k*, of the CBA porous concrete [33].

It is known that the thermal conductivity of concrete is strongly affected by the moisture content and the coarse aggregate in the concrete [34,35,36]. At the environmental temperature, this temperature is not enough to evaporate the internal water in the concrete or significantly change the moisture content in the concrete. Jansson [37] reported that the thermal conductivity of concretes measured by the TPS method were almost constant at temperatures from 20–100 °C. Then, the thermal conductivity was remarkably reduced from 100–600 °C. Therefore, it could be considered that thermal conductivity is insignificantly affected by the environmental temperature.

## 3. Results and Discussion

### 3.1. Unit Weight

**Figure 5:**
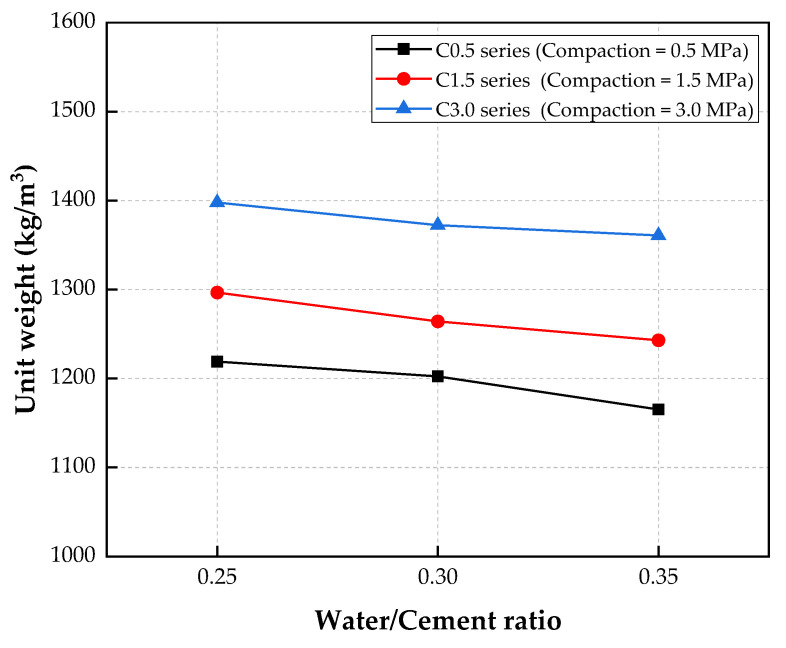
Unit weights of the porous concretes.

The influence of the water/cement ratio on the unit weight of the porous concrete is illustrated in Figure 5. This shows that increases in the water/cement ratios led to decreases in the unit weights for the three series. As shown in Table 5, for the C0.5 series, the unit weight decreased from 1219 kg/m^3^ to 1165 kg/m^3^ when the water/cement ratio was increased from 0.25 to 0.3. The same tendency was also observed for the C1.5 series; the unit weight of the C1.5 series decreased by 4.1% with the increasing water/cement ratio. Similarly, the unit weight of the C3.0 series decreased slightly, by 2.7%, with increases in the water/cement ratio. Accordingly, the unit weight of the CBA porous concrete was insignificantly affected by the water/cement ratio.

The porosity of porous concrete mainly depends on the aggregate particle size and aggregate type because there is just enough cement paste volume to cover the aggregate particles, and it cannot fill the voids between aggregates. Therefore, the water/cement ratio did not significantly affect the unit weight of the porous concrete. A similar tendency was also revealed by Costa et al. [38]. In their study, the unit weight of the porous concrete was reduced by 2.5% when the water/cement ratio was increased from 0.24 to 0.32.

Figure 5 shows the influence of the compaction on the unit weight of the porous concrete and indicates that the unit weight of the porous concrete was significantly affected by the compaction. The unit weight of the porous concrete increased significantly when the compaction level was increased from 0.5 to 3.0 MPa. With a water/cement ratio of 0.25, the unit weight of the C3.0-W25 specimen was 7.81% and 14.67% greater than those of the C1.5-W25 and C0.5-W25 specimens, respectively. With a water/cement ratio of 0.3, the unit weight of the porous concrete increased by 5.14% and 14.13% when the compaction level was increased from 0.5 MPa to 1.5 and 3.0 MPa, respectively. However, the standard deviations of the C0.5-W30 and C1.5-W30 series are high, and thus, a Student’s *t* test analysis between the two results was conducted. The analysis results showed that the *t_value_* of the two test results was 2.55, which was greater than the *t_α_* value of 2.15 from the Student’s *t* table. This result concluded that the two results were significantly different, and the compaction level affected the two series. Similarly, the unit weight of the C3.0-W35 specimen was greater than those of the C0.5-W35 and C1.5-W35 specimens. This was because the application of the compaction to the concrete specimens reduced the void content in the CBA porous concrete, and thus, the unit weight of the concrete increased, as presented in the test results.

Compared to the normal porous concrete, the CBA porous concrete showed a lower unit weight. Banevičiene et al. [39] cast normal porous concrete with different gravel coarse aggregate sizes, and the test results revealed that the unit weight of the normal coarse aggregate was in the range of 1800~2100 kg/m^3^. Meanwhile, the unit weight of the CBA porous concrete in this study was in the range of 1150~1400 kg/m^3^, which indicates that the unit weight of the porous concrete was significantly reduced with the inclusion of CBA.

### 3.2. Total Void Ratio

**Figure 6:**
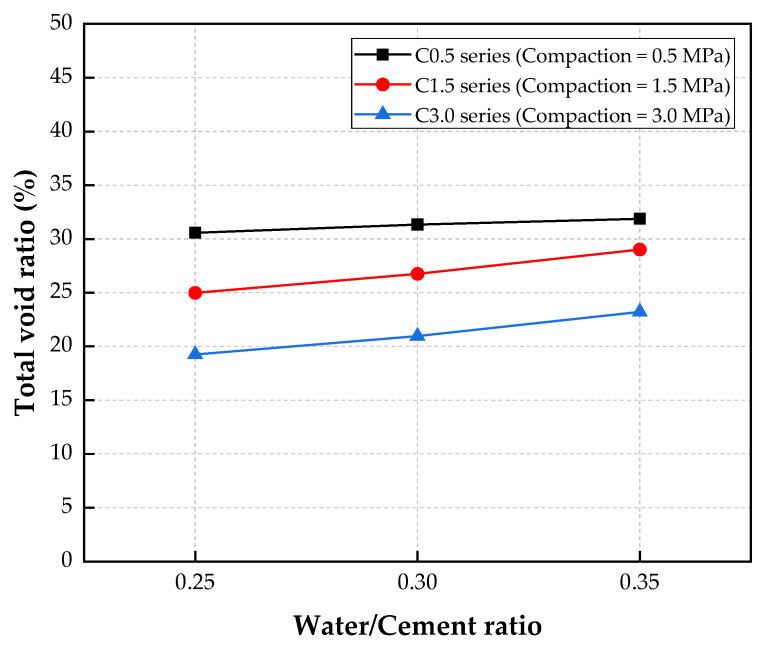
Total void ratios of the porous concrete samples.

The total void ratios observed for various water/cement ratios and compaction levels are presented in Figure 6.

For the C0.5 series of specimens, the total void ratio increased slightly from 30.6% to 31.9% when the water/cement ratio was increased from 0.25 to 0.35. However, for the C1.5 series, the total void ratio exhibited an increasing tendency with increases in the water-cement ratio. Specifically, the total void ratio increased significantly by 7.0% and 16.1% when the water/cement ratio was increased from 0.25 to 0.30 and 0.35, respectively. Additionally, for the C3.0 series, the total void ratios of the C3.0-W25, C3.0-W30, and C3.0-W35 specimens were 19.3%, 21.0%, and 23.2%, respectively.

Therefore, the test results showed that the total void ratios of the CBA porous concretes increased slightly with increases in the water/cement ratio. It could be assumed that an increase in the water/cement ratio in the mixture generated voids in the interface transition zone, which resulted in increases in the porosities of the porous concrete [38]. In a previous study by Debnath and Sarkar [27], they reported that increasing the water/cement ratio increased the workability of the porous concrete. However, the high workability resulted in a thinner cement paste coating the coarse aggregates, which increased the porosity of the porous concrete. A similar tendency was also observed in a study by Cui et al. [40] and Zhu et al. [41].

The influence of the compaction on the total void ratio can be found in Figure 6. The test results showed that the compaction of the concrete significantly decreased the total void ratio.

The total void ratio of the concrete specimen made with a compaction level of 3.0 MPa (C3.0 series) was always lower than those of concrete specimens treated with compaction levels of 0.5 (C0.5 series) and 1.5 MPa (C1.5 series), respectively. Specifically, for a water/cement ratio of 0.25, the total void ratio of the C3.0 specimen was 19.3%, which was 58.8% and 29.8% lower than those of the C0.5 and C1.5 specimens, respectively. Similarly, for a water/cement ratio of 0.30, the total void ratio of the specimen with a compaction of 3.0 MPa (C3.0-W30 specimen) was 27.6% and 49.5% lower than those of the C0.5-W30 and C1.5-W30 specimens, respectively. The standard deviation of the C0.5-W30 specimen was high, and thus, a Student’s *t* test analysis between the C0.5-W30 and C1.5-W30 series was performed. The *t_value_* between the two series was 2.85, which was higher than the *t_α_* of 2.18 from the Student’s *t* table. This indicates that there was a statistically significant difference between the C0.5-W30 and C1.5-W30 series and that the compaction levels influenced the two mixtures. In addition, for a water/cement ratio of 0.35, the total void ratio of the specimen with a compaction of 3.0 MPa (C3.0-W35 specimen) was 37.3% and 25.0% lower than those of the C0.5-W35 and C1.5-W35 specimens, respectively.

It was also reported that compaction directly affected the microstructures in porous concrete [42]. Increases in the compaction levels led to increases in the cement paste thickness, which covered the aggregate particles and reduced the void content in the porous concrete. Therefore, the total void ratio of the porous concrete decreased with the compaction.

### 3.3. Thermal Conductivity

**Figure 7:**
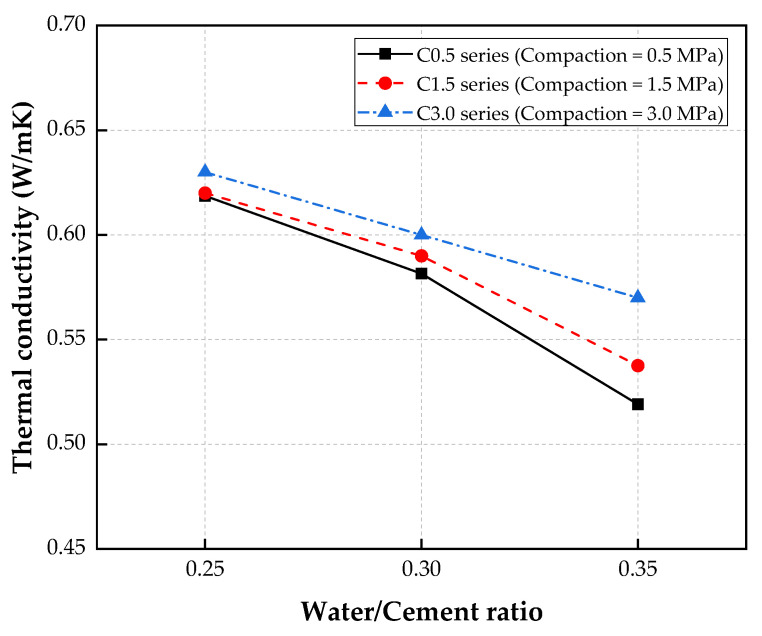
Thermal conductivities of porous concretes.

Figure 7 demonstrates the effects of the water/cement ratio and the compaction on the thermal conductivity of the porous concrete. The thermal conductivity of the porous concrete decreased when the water/cement ratio was increased. For the C0.5 series, the thermal conductivity decreased from 0.62 W/m∙K to 0.52 W/m∙K when the water/cement ratio was increased from 0.25 to 0.35. Similarly, for the C1.5 series, decreases in the thermal conductivity were also observed with increases in the water/cement ratio. Specifically, the thermal conductivities of the specimens with water/cement ratios of 0.30 and 0.35 were decreased by 5.1% and 15.3%, respectively, compared with that of the specimen made with a water/cement ratio of 0.25.

Finally, for the C3.0 series, the lowest thermal conductivity was also measured for the specimen with a water/cement ratio of 0.35 (C3.0-W35 specimen), while the C3.0-W25 and C3.0-W15 specimens showed greater values. It is known that the increase in the water/cement ratio results in higher capillary pores in cement paste at the hardening stage. In addition, the high water/cement ratio reduced the thickness of the cement paste coating the CBA, which led to an increase in the porosity of the CBA porous concrete. This phenomenon improved the thermal insulation of the CBA porous concrete.

Additionally, the influence of the compaction on the thermal conductivity of porous concrete is shown in Figure 7.

For specimens with a water/cement ratio of 0.25, the test results showed that the specimens made with the compaction levels of 0.5 MPa (specimen C0.5-W25) and 1.5 MPa (specimen C1.5-W25) exhibited similar thermal conductivities, but the thermal conductivity of the specimen subjected to a compaction level of 3.0 MPa (specimen C3.0-W25) was approximately 1.8% greater than that of specimen C0.5-W25. Among the specimens with a water/cement ratio of 0.30, specimen C3.0-W30 also showed the greatest thermal conductivity at 0.60 W/m∙K, which was 3.2% and 1.7% higher than those of specimens C0.5-W30 and C1.5-W30, respectively. Additionally, for specimens with a water/cement ratio of 0.35, the thermal conductivity of the specimen made with a compaction level of 3.0 MPa (C3.0-W35) was higher than that of any other specimen.

Therefore, increases in the compaction level led to decreases in the porosity of the CBA porous concrete, which resulted in decreases in the thermal conductivity. Thermal conductivity and porosity are inversely proportional, which was reported previously by Chen et al. [43]. In their study, the thermal conductivity of the porous concrete decreased as the porosity of the concrete increased. Chindaprasirt et al. [44] reported that the thermal properties of porous concrete were primarily contributed by the void content from the porous concrete and the porosity of the aggregates. By increasing the compaction, the CBA particles were compacted, which reduced the void content in the porous concrete, and the concrete became denser.

In addition, the use of CBA as the coarse aggregate in porous concrete improved the thermal insulation characteristics. Compared to that of conventional porous concrete, the thermal conductivities of the CBA porous concrete showed lower values, while those of conventional porous concrete was in the range of 1.80–2.02 W/m∙K [43]. Torkittikul et al. [45] investigated the remarkable effects of CBA on the thermal insulation capacity of concrete and mortar. The test results of this study also indicated that the addition of CBA to the mixtures significantly improved the thermal insulation characteristics of the concrete. Yang and Park [15] reported that because of the pore structure in CBA particles, the use of CBA in concrete significantly improved the thermal properties of the concrete.

### 3.4. Scanning Electron Microscope (SEM) Analysis

**Figure 8:**
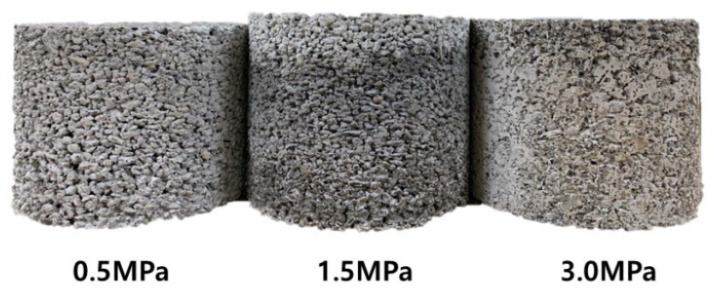
Surfaces of porous concrete samples subjected to various compaction levels.

The surfaces of porous concrete specimens prepared with three different compaction levels are shown in Figure 8. This shows that the porous concrete made with a higher compaction level was denser. The void distances between the aggregates on the surface of the concrete gradually decreased when the compaction level was increased.

**Figure 9:**
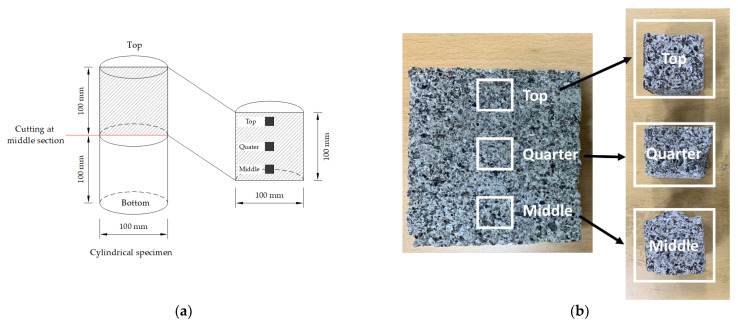
Extraction method. (**a**) Illustration of extraction method; (**b**) Extracted pieces.

In addition, to consider the effect of the compaction level at different positions along the depth of the specimen, scanning electron microscopy (SEM) analysis was performed. To obtain the examined pieces, the cylindrical specimen was cut in half. Then, three different pieces at different positions with dimensions of 20 mm × 20 mm × 10 mm were extracted from the half top of the cylindrical specimens, as shown in Figure 9.

**Figure 10:**
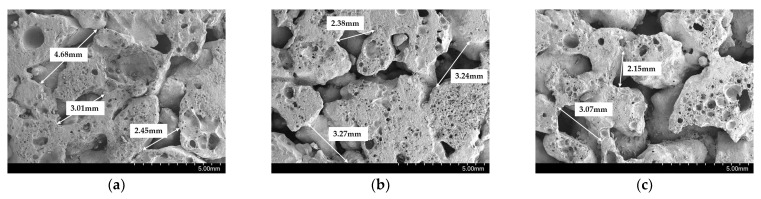
SEM images of the CBA porous concrete at different positions. (**a**) Top position; (**b**) Quarter position; (**c**) Middle position.

Figure 10 reveals the SEM results at different positions of the porous CBA specimen. This shows that the impact of the compaction level was different at the different positions along the depth of the specimen. As shown in the figure, the distance between the particles or the voids in the CBA concrete decreased along the specimen depth. This reveals that the cohesive strength between the particles was improved by applying compaction. However, the CBA particles tended to expand after the compaction was released. Therefore, different impacts at different positions along the depth of the specimen by the compaction were observed.

## 4. Relationships among Test Results

### 4.1. Relationship between Unit Weight and Porosity

**Figure 11:**
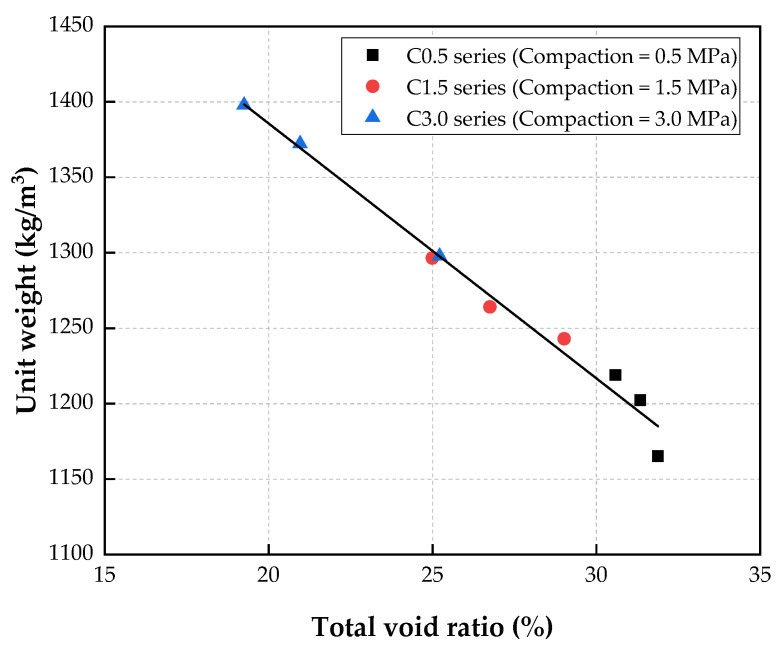
Relationship between unit weight and total void ratio.

The relationship between the unit weight and the total void ratio is shown in Figure 11. This indicates that the unit weight was inversely proportional to the total void ratio of the CBA porous concrete.

As indicated by regression analysis, the relationship between the unit weight and the total void ratio under various compaction levels was expressed exponentially by the following equation:

(2) $$U=1723.53-16.89\times V\;\;\;\;\;\;\;\;R^{2}=0.9834$$

where *U* is the unit weight (kg/m^3^), and *V* is the total void ratio (%) of the CBA porous concrete. The coefficient of determination (*R*^2^) for this equation was close to one, which indicated that the equation could be used to predict the unit weight precisely by using the total void ratio of the porous concrete.

### 4.2. Relationship between Thermal Conductivity and Unit Weight

**Figure 12:**
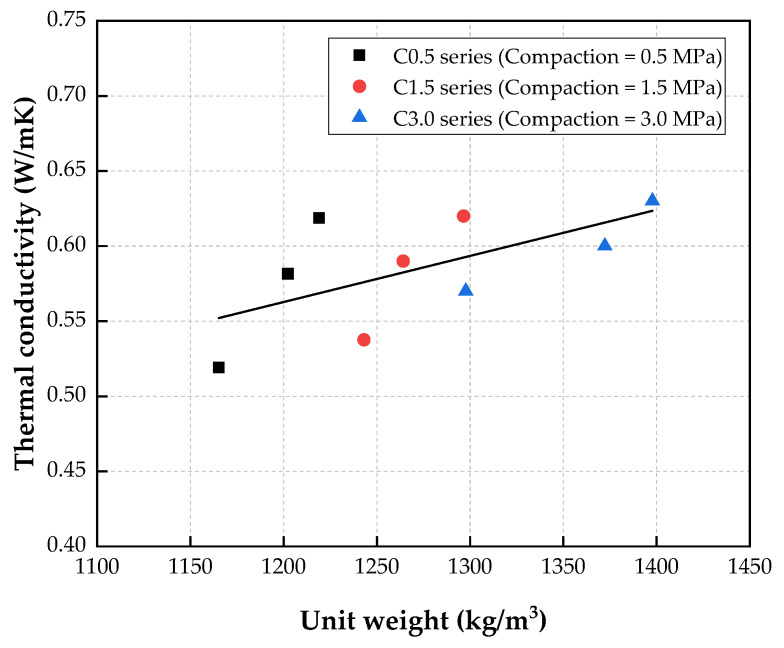
Relationship between thermal conductivity and unit weight.

Figure 12 illustrates the relationship between the thermal conductivity and unit weight of CBA porous concrete. As shown in this figure, the thermal conductivity of the porous concrete measured in this study presented an overall increasing tendency as the unit weight of the concrete increased.

The proposed equation relating the thermal conductivity of porous concrete to the unit weight is as follows:

(3) $$K=0.1942+0.0003\times U\;\;\;\;\;\;\;\;R^{2}=0.3876$$

where *K* is the thermal conductivity (W/m∙K), and *U* is the unit weight (kg/m^3^) of the porous concrete.

The coefficient of determination (*R*^2^) for this equation was low because the measured values deviated from the regression line due to a lack of measurements. Accordingly, the accumulation of more measured data would increase the coefficient of determination.

### 4.3. Relationship between Thermal Conductivity and Total Void Ratio

**Figure 13:**
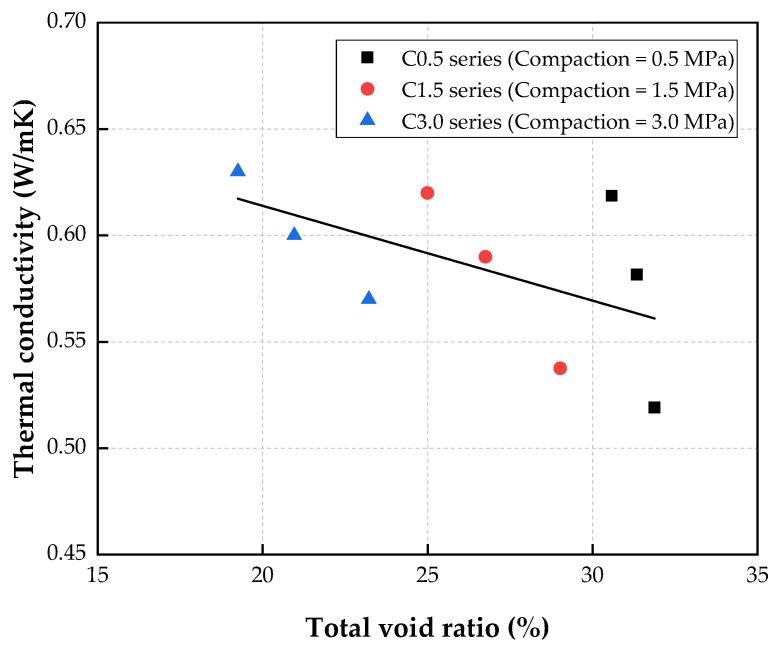
Relationship between thermal conductivity and total void ratio.

Figure 13 demonstrates the relationship between the thermal conductivity and the total void ratio of the CBA porous concrete. This figure shows that the overall thermal conductivity of the porous concrete decreased as the total void ratio increased.

Based on the test results of this study, the linear equation predicting the relationship of the thermal conductivity for porous concrete with the total void ratio is as follows:

(4) $$K=0.7031-0.0045\times V\;\;\;\;\;\;\;\;R^{2}=0.2974$$

where *K* is the thermal conductivity (W/m∙K), and *V* is the total void ratio (%) of the porous concrete.

The plot of the thermal conductivity versus the total void ratio exhibited a low coefficient of determination, which was due to high variability in the test results. However, more measured data would likely increase the coefficient of determination and, thus, improve the accuracy of the prediction.

## 5. Conclusions

The thermal conductivity of CBA porous concrete was investigated in this study. The effects of the water/cement ratio and compaction level on the unit weight, total void ratio, and thermal conductivity of CBA porous concrete were analyzed. In addition, statistical analysis was conducted to ensure a statistically significant difference between the results. Based on the extensive test results, the following conclusions can be drawn from this study:

1. The unit weight of CBA porous concrete was slightly affected by the water/cement ratio. An increase in the water/cement ratio from 0.25 to 0.35 led to decreases in the unit weight of the porous concrete ranging from 3.0 to 5.0%. Moreover, the compaction level significantly increased the unit weight of the CBA porous concrete by 5.0 to 15.0%. In addition, the statistical results indicated that the compaction levels had different influences on each mixture.
2. The test results revealed that the total void ratio of the CBA porous concrete increased as the water/cement ratio increased, but this increase was not significant. However, the increases in the total void ratios of CBA porous concrete ranged between 7.0% and 17.0% with the increasing compaction level. It was also observed that the compaction dramatically reduced the porosity of the concrete. Statistical analysis was also carried out and revealed the statistically significant differences between the results.
3. Regarding the effect of the water/cement ratio on the thermal conductivity of the CBA porous concrete, the thermal conductivity was significantly reduced by increases in the water/cement ratio. The thermal conductivity of the CBA porous concrete decreased by 4% to 16% as the water/cement ratio was increased from 0.25 to 0.35.
4. The test results also revealed that the thermal conductivity of the CBA porous concrete decreased by 0.2% to 9.6% with increases in the compaction level. This implied that the thermal insulation of the CBA porous concrete was not significantly affected by the compaction levels used in this study, but the other properties were significantly improved.
5. Finally, the relationships between the thermal conductivity and the mechanical properties of the porous concrete were estimated. By using the relationship curves, the tendency of thermal conductivity relating to the unit weight and total void ratios could be determined.

This study primarily focused on the thermal properties of CBA porous concrete. The experiment on the permeability and durability of the CBA concrete was out of the scope of the study. However, the permeability and durability of the CBA porous concrete will be investigated in future studies.

## Figures and Tables

**Table 1:** Sieve test of CBA.

Nominal Diameter	Remaining Weight	Remaining Ratio	Cumulative Remaining Ratio	Passing Weight	Passing Ratio
(mm)	(g)	(%)	(%)	(g)	(%)
10.0	0.00	0.00	0.00	249.05	100.00
5.0	2.29	0.92	0.92	246.76	99.08
2.5	188.03	75.50	76.42	58.73	23.58
1.2	56.95	22.87	99.29	1.78	0.71
0.6	0.32	0.13	99.41	1.46	0.59
0.3	0.03	0.01	99.43	1.43	0.57
0.12	0.10	0.04	99.47	1.33	0.53
pan	1.33	0.53	100.00	0.00	0.00
Total	249.05	100		Fineness modulus	4.75

**Table 2:** Physical properties of CBA.

Hybrid CBA Size	SSD Density	Oven-Dried Density	Absorption
	(g/cm^3^)	(g/cm^3^)	(%)
2.5~5.0 mm (80%) + 1.2~2.5 mm (20%)	1.73	1.61	7.75

**Table 3:** Chemical compositions of CBA and OPC.

Component	CBA	OPC
	(%)	(%)
SiO_2_	55.75	17.65
Al_2_O_3_	26.15	4.64
Fe_2_O_3_	7.51	3.41
CaO	3.95	64.90
Na_2_O	0.73	0.21
MgO	1.12	3.51
K_2_O	1.19	1.15
SO_3_	0.74	3.71
LOI	2.43	0.73

Note: OPC: ordinary Portland cement.

**Table 4:** Mixing proportions of CBA porous concrete.

No	Mixture	w/c Ratio	Unit Weight (kg/m^3^)				CA	Compaction
			Water	OPC	CBA			MPa
					1.2~2.5 mm	2.5~5.0 mm		
1	C0.5-W25	0.25	110.0	440.0	246.9	964.9	38.5	0.5
2	C1.5-W25		110.0	440.0	246.9	964.9	38.5	1.5
3	C3.0-W25		110.0	440.0	246.9	964.9	38.5	3.0
4	C0.5-W30	0.30	110.0	366.7	255.0	996.7	38.5	0.5
5	C1.5-W30		110.0	366.7	255.0	996.7	38.5	1.5
6	C3.0-W30		110.0	366.7	255.0	996.7	38.5	3.0
7	C0.5-W35	0.35	110.0	314.3	260.8	1019.5	38.5	0.5
8	C1.5-W35		110.0	314.3	260.8	1019.5	38.5	1.5
9	C3.0-W35		110.0	314.3	260.8	1019.5	38.5	3.0

Note: OPC: ordinary Portland cement, CBA: coal bottom ash, and CA: cohesive agent.

**Table 5:** Properties of CBA porous concrete.

Mixture	Unit Weight		Total Void Ratio		Thermal Conductivity	
	(kg/m^3^)		(%)		(W/m∙K)	
	Mean	S.D.	Mean	S.D.	Mean	S.D.
C0.5-W25	1219	22	30.6	1.8	0.62	0.01
C1.5-W25	1297	31	24.9	2.8	0.62	0.01
C3.0-W25	1398	51	19.3	2.0	0.63	0.02
C0.5-W30	1202	58	31.3	3.9	0.58	0.01
C1.5-W30	1264	37	26.8	2.8	0.59	0.02
C3.0-W30	1372	34	20.9	1.7	0.60	0.02
C0.5-W35	1165	23	31.9	1.9	0.52	0.01
C1.5-W35	1243	30	29.0	2.0	0.54	0.03
C3.0-W35	1361	50	23.2	3.1	0.57	0.01

Notes: S.D.: standard deviation.

## Data Availability

The data used to support the findings in this study are available from the corresponding author upon request.
